# Evaluation of interfractional variation of the centroid position and volume of internal target volume during stereotactic body radiotherapy of lung cancer using cone‐beam computed tomography

**DOI:** 10.1120/jacmp.v17i2.5835

**Published:** 2016-03-08

**Authors:** Yanan Sun, Hong Ge, Siguo Cheng, Chengliang Yang, Qianqian Zhu, Dingjie Li, Yuan Tian

**Affiliations:** ^1^ Department of Radiation Oncology The Affiliated Cancer Hospital of Zhengzhou University 127 Dongming Road Zhengzhou 450008 Henan Province China; ^2^ Red Cross Blood Center of Henan 9 Tongle Road Zhengzhou 450053 Henan Province China; ^3^ Department of Radiation Oncology Cancer Hospital Chinese Academy of Medical Science 18#, Panjiayuan South Road, Chaoyang District Beijing China

**Keywords:** stereotactic body radiation therapy, internal target volume, tumor regression, interfractional variation of tumor centroid position, cone‐beam computed tomography

## Abstract

The purpose of this study was to determine interfractional variation of the centroid position and volume of internal target volume (ITV) during stereotactic body radiation therapy (SBRT) of lung cancer. From January 2014 to August 2014, a total of 32 patients with 37 primary or metastatic lung tumors were enrolled in our study. All patients received SBRT treatment in 4–5 fractions to a median dose of 48 Gy. Both 3D CT and 4D CT scans were used for radiotherapy treatment planning. 3D CBCT was acquired prior to treatment delivery to verify patient positioning. A total of 163 3D CBCT images were available for evaluation. 3D CBCT scans acquired for verification were registered with simulation CT scans. The ITVs were contoured on all verification 3D CBCT scans and compared to the initial gross target volume (GTV) or ITV in treatment planning system. GTV was based on 3D CT while ITV was based on both 3D CT and 4D CT. To assess the interfractional variation of ITV centroid position, we used vertebrae body adjacent to the tumor as reference point when performing the registration procedure. To eliminate the effect of time on tumor volume between simulation CT scan and the first fraction, the interfractional variation of ITV was evaluated from the first fraction to the last fraction. The overall 3D vector shift was 4.4±2.5 mm (range: 0.4−13.8 mm). The interfractional variation of ITV centroid position in superior‐inferior, anterior‐posterior, and left‐right directions were −0.7±2.7 mm, −1.4±3.4 mm, and −0.5±2.2 mm, respectively. No significant difference was observed between three directions (p=0.147). Large interfractional variations (≥5 mm) were observed in 12 fractions (9.3%) in superior‐inferior direction, 24 fractions (18.6%) in anterior‐posterior direction, and 5 fractions (3.9%) in left‐right direction. No time trend of tumor volume change measured in 3D CBCT was detected during four fractions (p=0.074). A significant (p=0.010) time trend was detected when evaluating the time trend of ITV change during 5 fractions and diameter was found to be significantly correlated with the ITV change (p=0.000). ITV did not show significant regression during SBRT treatment, but interfractional variation in the ITV centroid position was observed, especially in anterior‐posterior direction. An isotropic margin of 7 mm around ITV might be necessary for adequate coverage of interfractional variation of ITV centroid position, but only in case no soft tissue‐based setup is performed during SBRT treatment.

PACS number(s): 87.55.dk

## I. INTRODUCTION

Stereotactic body radiotherapy (SBRT) offers a genuine alternative to surgery for patients with early stage non‐small cell lung cancer (NSCLC) who cannot tolerate surgery due to coexisting illnesses (i.e., coronary heart disease, insufficiency pulmonary function), or who refuse to take surgery.^1^, ^2^, ^3^ It is reported that local control rates can reach 85%−90% after SBRT for early stage lung cancer, which is almost equal to surgery alone.^1^, ^2^ Up to now, the clinical application of SBRT has been extended to oligometastatic lung tumors and other types of malignancies, such as liver cancer and pancreatic cancer.^4^, ^5^, ^6^ However, the implementation of SBRT technique is a great concern for its single high dose irradiated to target volume within a short time. So, accurate localization and irradiation of tumors appears to be extremely important for SBRT technique. Lung tumor motion due to breathing not only causes partially missing of tumor volume but also unnecessary irradiation of normal adjacent tissues. To solve this problem, several measures have been taken: encompassing tumor motion range with four‐dimensional computed tomography (4D CT);^(7)^ using abdominal compression or taking breath‐hold technique;^8^, ^9^ and using tracking technique to deliver respiratory gated radiotherapy.^(10)^ As a relatively convenient method, 4D CT scan is currently used for radiotherapy simulation of lung tumors in our institution. It can cover the motion trajectory of lung tumors, thereby make it available to obtain the internal target volume (ITV) in the treatment planning system.^(11)^

In the past decades, cone‐beam computed tomography (CBCT) has been used for position verification as it can acquire CT images at all phases of the respiration cycle.^(12)^ Direct comparison between CT images obtained by 4D CT and CBCT could reduce the effect of tumor motion induced by respiration in three dimensions, because both 4D CT and CBCT scans can cover the motion trajectory of tumors in different imaging modalities. However, the respiratory pattern is not invariant through the whole process of radiotherapy and there are many other factors influencing the position and volume of lung tumors, such as tumor shrinkage, coexisting disease, and heartbeat.^13^, ^14^ Though SBRT treatment is expected to terminate within two weeks, interfractional tumor position variation also should be considered due to potential impact of its high single dose on normal tissues. Thus, the aim of our study is to evaluate the interfractional variation in ITV centroid position and volume during SBRT using 3D CBCT.

## II. MATERIALS AND METHODS

A retrospective analysis was carried out for lung cancer patients who underwent SBRT from January 2014 to August 2014. Patients qualified for SBRT must satisfy the following criteria: tumor diameter <5 cm, tumors located at peripheral lung parenchyma, early stage NSCLC (T1 or T2 stage) or metastatic lung tumor (<5 tumors in one patient), no contraindication to radiotherapy. Informed consent document was necessary for each patient before SBRT treatment. Finally, 32 patients with 37 peripheral lung tumors were available in our study.

### A. Computed tomography scan and image reconstruction

All patients were immobilized with evacuated bags in head‐first supine position with arms above head. Treatment simulation was conducted on a 16‐slice CT scanner (Brilliance CT; Big Bore; Philips Healthcare, Andover, MA). Related scan parameters were as follows: 120 kV, 350 mAs, pitch 0.938, slice thickness 3 mm. For 26 patients, both three‐dimensional computed tomography (3D CT) and four‐dimensional computed tomography (4D CT) were used for treatment planning. So, treatment simulation was separated into two stages. Patients were instructed to lie down in the same gesture to make sure there were no dislocations of patient position and no gap in the evacuated bags. Acting as sensor, an elastic strap was placed along the lower rib cage. Firstly, patients were instructed to breathe normally during the scan. Loversol injection was used as intravenous contrast for the free‐breathing helical 3D CT scan. Scanning scope incorporated the region between the superior border of the fourth cervical vertebra and the bottom of the lung. Secondly, 4D CT scan was carried out without contrast. Patients were also instructed to breathe normally. The elastic strap was used to monitor the respiratory cycle, which allowed the generation of a waveform signal in the display screen. Once the waveform became stable, the 4D CT scan could start. Scan range covered the region between the apex of lung and the bottom of lung. After scan, 10 phases of 4D CT images were reconstructed based on the respiratory signal from the sensor in the Brilliance Big Bore CT workstation. Ten phases were defined from 0% to 90%; 0% phase represented the peak inspiration phase and 50% represented the peak expiration phase. In addition, maximum intensity projection (MIP) and average intensity projection (AIP) were also reconstructed based on 10 phases of CT images. For six patients, only 3D CT scans were obtained for treatment planning. Finally, all CT images’ data were transferred to the Eclipse treatment planning system (Varian Medical Systems, Inc., Palo Alto, CA).

### B. Treatment planning

**Figure 1:**
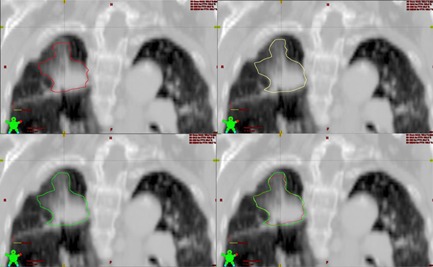
Example of the ITV combined definition of an early stage non‐small cell lung cancer. The delineations of tumor volume were projected to AIP images. The red line represents GTV 3D, the yellow line represents ITVmip, and green line represents ITVcombined, which is the combination of GTV 3D and ITVmip. We can see from the upper‐left picture that GTV 3D cannot encompass the full range of tumor volume, but ITVmip can cover the tumor volume to a large extent.

In our study, different methods were employed to contour the target volume of lung tumors. For patients who underwent both 3D CT and 4D CT scans, tumor volume contouring referred to our previous study.^(15)^ Firstly, gross target volume (GTV) was delineated on the 3D CT images, which was labeled as GTV‐3D. Both lung window (window width 1000/window location−650) and mediastinum window (window width 350/window location 40) were used to identify their location and their relationship with adjacent structures. This is especially necessary for those tumors which abutted to mediastinum, diaphragm, or chest wall. Then, reconstructed MIP images which included the motion trajectory of the tumor were used to delineate the internal target volume (ITVmip). Finally, combined internal target volume (ITVcombined) was obtained by combination of GTV‐3D and ITVmip. Figure 1 shows one example of the ITVcombined definition. No clinical target volume (CTV) margin was added to ITV and an isotropic margin of 5 mm was added in three directions for PTV, referring to the study performed by Takahashi W, et al.^(16)^ Compared with AIP images, free‐breathing images were more prone to significant image artifacts and MIP images may mistakenly estimate the target volume when the tumor is closer to the denser tissue, so AIP is deemed favorable for planning and dose calculation for lung SBRT.^(17)^ Thus, in our study, the dose calculation of radiotherapy planning was performed on AIP images and AIP images were sent to the workstation of the accelerator for setup. For patients who underwent 3D CT scan only, GTV was delineated according to the method mentioned above. For the margin of PTV, 10 mm was added to craniocadual direction and 5 mm was added to both anterior‐posterior and left‐right directions. Two radiation oncologists with extensive experience in lung SBRT were required to reach a consensus when evaluating the delineation extent of the target volume. Volumetric‐modulated arc therapy (VMAT) technique was adopted to design the radiotherapy planning. Prescription dose was required to encompass 95% of PTV region.

### C. Delivery of radiotherapy

Patients waited no more than seven days before the beginning of radiotherapy. All patients were treated by TrueBeam SN1403 accelerator (Varian Medical Systems) equipped with cone‐beam computed tomography (CBCT), which can obtain the breathing movement information over several respiration cycles. The patients were instructed to lie down in the evacuated bags in the same posture as during CT simulation, and the position was adjusted referring to red lines marked in patients’ chest wall before performing simulation CT. The couch was moved based on the data calculated by treatment planning system and then a 3D CBCT scan was carried out. The patients were required to maintain stable breathing during 3D CBCT scan. No respiration gating system (RPM) was used when carrying out 3D CBCT scan, so the images could not be divided into 10 phases according to the respiration signal. 3D CBCT scan was compared with 3D CT (for those patients who underwent 3D CT scan only) or AIP images of planning 4D CT (for those patients who underwent both 3D CT and 4D CT scans) for evaluating the setup error before treatment of each fraction. Soft‐tissue‐based image registration was used to calculate the setup error. Generally, automatic registration was employed for match. If automatic registration could not achieve ideal match, manual registration would be employed. The lung lesion visualized in 3D CBCT was aligned to reference planning CT (3D CT or AIP image of planning 4D CT) by matching the central region with high contrast. Not until the setup error was less than 2 mm could the radiotherapy be started. If the setup error of more than 2 mm persisted, patients would be required to adjust their body position to receive another 3D CBCT scan. Because of the single high dose of SBRT, patients were required to receive radiotherapy on alternative days. The duration of SBRT treatment was no more than two weeks. Finally, a total of 163 3D CBCT images were available for evaluation.

### D. Data acquisition of interfractional variation of tumor centroid position and volume

All patients underwent 3D CBCT scan before treatment. The acquired 3D CBCT images were registered to the reference CT images (AIP images or 3D CT images). In order to eliminate the time effect between the simulation and the 1st fraction of SBRT, we evaluated the volume trend of ITV from the first fraction to the last fraction. So, all tumors were available for evaluating interfractional variation of tumor volume. Since the impact of respiration was not taken into account in patients who underwent 3D CT scan only, ITV was not obtainable for treatment planning, so, interfractional variation of ITV centroid position could not be calculated out for this group of patients. Those patients who underwent both 3D CT scan and 4D CT scan were included. Though several setup methods, including soft‐tissue‐based setup and bony‐structure‐based setup, were adopted in clinical practice, bony‐structure‐based registration is the basic assumption for the evaluation of interfractional variation of ITV centroid position. Because the pattern and amplitude of breathing will vary from fraction to fraction and it will have an impact on the imaging results of CBCT,^(18)^ there are still many uncertainties when registering CBCT with 4D CT using soft‐tissue‐based setup method and it will help determining ITV‐PTV margin in case no soft‐tissue‐based setup is available during SBRT treatment. However, bony structure is relatively stable and identifiable in CBCT images. In our study, we used vertebrae adjacent to the tumor as reference point when performing the registration procedure. ITVs were delineated in each 3D CBCT images based on the lung window and mediastinum window mentioned in treatment planning, section B, above. A single radiation oncologist (Sun Y) was required to perform the delineation procedure under the supervision of two senior radiation oncologists with expertise in SBRT. The Eclipse treatment planning system can calculate the ITV centroid position automatically, so the interfractional variation of each ITV centroid position of treatment with reference to their ITVs in treatment planning was figured out with the help of Eclipse treatment planning system. The ITV centroid position was expressed in three dimensions: superior‐inferior, anterior‐posterior, and left‐right directions. Additionally, the 3D vector shift was computed by taking the root sum square of interfractional variation in three directions.

### E. Baseline characteristic of patients and tumors

In our study, 32 patients with 37 tumors were available. Of them, 21 patients were male and 11 patients were female. The median age of the cohort was 70 years (range: 47 to 86 years). The median diameter of cross‐sectional area of the tumor was 2.20 cm (range: 0.88−5.14 cm). Of all patients, 20 patients were diagnosed as primary lung cancer by bronchoscope or percutaneous transthoracic lung biopsy, of which 9 patients were diagnosed as squamous cell lung cancer and 11 patients lung adenocarcinoma; 3 patients had no biopsy for poor pulmonary function or cardiovascular disease, but their clinical examination, including PET‐CT, was highly suggestive of primary lung cancer; 8 patients were diagnosed as metastatic lung cancer, originating from (for example) esophagus or rectum. Of all tumors, 10 were located in the upper lobe of left lung, 12 located in upper or middle lobe of right lung, 8 located in the lower lobe of left lung, and 7 located in lower lobe of right lung. Four types of SBRT regimens were adopted for SBRT treatment: 50 Gy in 5 fractions for 13 tumors; 48 Gy in 4 fractions for 14 tumors; 40 Gy in 4 fractions for 8 tumors; 40 Gy in 5 fractions for 2 tumors.

### F. Statistical analysis

All statistical analyses were performed by SPSS Statistics V22.0 (IBM Corp., Armonk, NY). Quantitative data were expressed as mean±standard deviation (SD). Generalized estimating equation was used to examine the time trend of ITV change and 3D vector shift, with diameter and location as predictive factors. Location was classified as two groups: upper lobe and lower lobe. Diameter was classified into two groups: diameter <3 cm and diameter >3 cm.

## III. RESULTS

### A. Interfractional variation of ITV centroid position

In our study, both 3D CT scan and 4D CT scans were used for radiotherapy simulation for 26 patients, but 3D CT scan only was used for six patients. ITV could not be obtained in these six patients, so they were not included when evaluating the interfractional variation of ITV centroid position. In addition, severe humpback was detected in one patient, so the coordinate system of ITV was not the same with normal individuals. Finally, 30 tumors were available for evaluating the interfractional variation of ITV centroid position.

**Figure 2:**
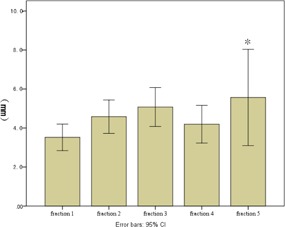
3D vector shift of tumor centroid position with elapsed time. The height of each bar represents the mean 3D vector shift of each fraction. Vertical lines represent the 95% error bars. *Because 15 tumors received the 5th fraction of SBRT, the fifth bar represents only this group of patients.

**Figure 3:**
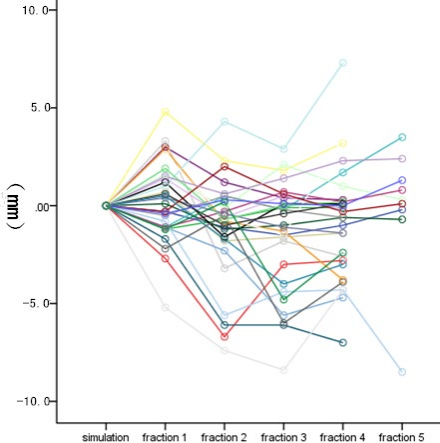
The interfractional variation of ITV centroid position in SI direction. Each color represents the interfraction variation of tumor position of one patient with elapsed time. The ITV centroid position of simulation was used as reference.

**Figure 4:**
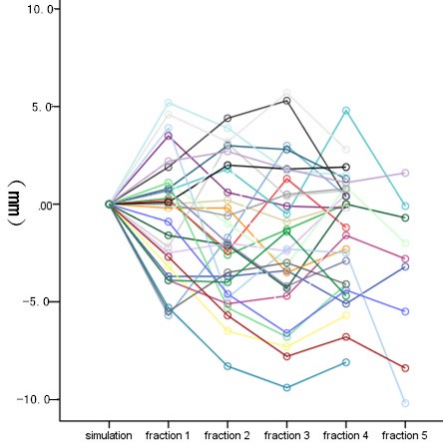
The interfractional variation of ITV centroid position in the AP direction. Each color represents the interfraction variation of tumor position of one patient with elapsed time. The ITV centroid position of simulation was used as reference.

**Figure 5:**
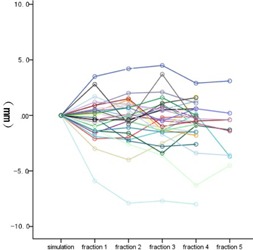
The interfractional variation of ITV centroid position in LR direction. Each color represents the interfraction variation of tumor position of one patient with elapsed time. The ITV centroid position of simulation was used as reference.

The overall 3D vector shift was 4.4±2.5 mm (range: 0.4−13.8 mm). No time trend of 3D vector shift was detected from the 1st fraction to the 5th fraction (p=0.483). Figure 2 shows the 3D vector shift with elapsed time. The interfractional variation of ITV centroid position in superior‐inferior (SI), anterior‐posterior (AP), and left‐right (LR) directions was −0.7±2.7 mm, −1.4±3.4 mm, and −0.5±2.2 mm, respectively. A relative higher variation was observed in AP direction, but it was not significantly different with the other two directions (p=0.147). Three line charts (Figs. 3, 4, and 5) show the interfractional variation of ITV centroid position in three directions.

Of all 129 fractions, absolute interfractional variation of ITV centroid position more than 5 mm was observed in 12 fractions (9.3%) in SI direction, 24 fractions (18.6%) in AP direction, and 5 fractions (3.9%) in LR direction. When setting 7 mm as the cutoff point, the corresponding values were 6 fractions (4.7%) in SI direction, 8 fractions (6.2%) in AP direction, and 3 fractions (2.3%) in LR direction. When setting 10 mm as the cutoff point, the corresponding values were 1 fraction (0.8%) in SI direction, 1 fraction (0.8%) in AP direction, and 0 fraction (0%) in LR direction. Overall, absolute interfractional variation (any direction) more than 5 mm, 7 mm, and 10 mm was observed in 37 fractions (28.7%), 12 fractions (9.3%), and 1 fraction (0.8%), respectively.

Based on the formula deduced by Zhang et al,^(19)^ the 1D asymmetric expansion for single fraction is: $C_{i}=2.33^{*}(\text{SD})$. This means that the margin added to each direction should be the standard deviation of the interfractional variation of ITV centroid position multiplied by 2.33. Similarly, the ITV‐PTV margins for AP, SI, and LR directions in our study theoretically should be 7.9 mm, 6.2 mm, and 5.0 mm, respectively.

### B. Interfractional variation of ITV

All patients underwent 3D CBCT scan for verification before each fraction of SBRT and the ITV was calculated from the first fraction to the last fraction, so all patients were available when evaluating ITV change during SBRT treatment.

Of all tumors, 16 tumors underwent 5 fractions of SBRT treatment, while 21 tumors underwent 4 fractions. The median ITV obtained from the 1st fraction of 3D CBCT was $8.70\;{\text{cm}}^{3}$ (range: $0.40-60.60\;{\text{cm}}^{3}$) for all tumors, $15.35\;{\text{cm}}^{3}$ (range: $2.00-60.60\;{\text{cm}}^{3}$) for 16 tumors that underwent 5 fractions, and $6.50\;{\text{cm}}^{3}$ (range: $0.40-22.10\;{\text{cm}}^{3}$) for 21 tumors that underwent 4 fractions. The ITVs of tumors treated in 5 fractions were larger than those treated in 4 fractions (p<0.000). The median ITV obtained from the following fractions of 3D CBCT were $7.80\;{\text{cm}}^{3}$ (range: $0.50-58.60\;{\text{cm}}^{3}$), $7.30\;{\text{cm}}^{3}$ (range: $0.60-56.20\;{\text{cm}}^{3}$), $6.30\;{\text{cm}}^{3}$ (range: $0.60-41.8\;{\text{cm}}^{3}$), and $17.40\;{\text{cm}}^{3}$ (range: $1.60-38.90\;{\text{cm}}^{3}$), respectively. Because each tumor underwent at least 4 fractions of SBRT treatment, we evaluated the time trend of ITV change during four fractions only. The result of generalized estimated equation showed that no time trend of ITV change was detected during four fractions (p=0.074). However, when evaluating the time trend of ITV change during 5 fractions, a significant time trend was detected (p=0.010). Both location and diameter were included as predictors, but only diameter was found to be significantly associated with the ITV change (p=0.000).

Thus, we evaluated the percentage of ITV shrinkage compared with ITV obtained from the first 3D CBCT image according to the stratification factor of diameter. The formula can be expressed as follows: the fraction of ITV shrinkage equals to (ITVn−ITV1)/ITV1 multiplying 100% (2<n<5; n represents the fraction order of SBRT). The percentage of ITV shrinkage in all tumors and tumors with different diameters are enumerated in Table 1, Table 2, and Table 3.

**Table 1:** The percentage of ITV shrinkage in all tumors

*Fraction Percentage*	*Mean*	*SD*	*Minimum Value*	*Maximum Value*
pre‐fraction 2	−2.8%	16.0%	−50.0%	28.6%
pre‐fraction 3	−2.2%	24.2%	−87.2%	31.5%
pre‐fraction 4	4.0%	28.1%	−81.7%	59.5%
pre‐fraction 5^a^	5.2%	20.7%	−53.2%	35.8%

a ^a^ Only 15 tumors were available for evaluating the percentage of ITV shrinkage before the 5th fraction of SBRT.

**Table 2:** The percentage of ITV shrinkage in tumors with diameter >3 cm

*Fraction Percentage*	*Mean*	*SD*	*Minimum Value*	*Maximum Value*
pre‐fraction 2	−2.3%	11.3%	−27.0%	10.3%
pre‐fraction 3	−2.2%	17.6%	−48.7%	15.8%
pre‐fraction 4	7.9%	28.4%	−49.1%	59.5%
pre‐fraction 5^a^	4.1%	21.1%	−32.4%	35.8%

a ^a^ Only 15 tumors were available for evaluating the percentage of ITV shrinkage before the 5th fraction of SBRT.

**Table 3:** The percentage of ITV shrinkage in tumors with diameter <3 cm

*Fraction Percentage*	*Mean*	*SD*	*Minimum Value*	*Maximum Value*
pre‐fraction 2	−3.1%	18.0%	−50.0%	28.6%
pre‐fraction 3	−2.1%	27.3%	−87.2%	31.5%
pre‐fraction 4	2.2%	28.3%	−81.7%	43.1%
pre‐fraction 5^a^	5.9%	21.4%	−53.2%	33.7%

a ^a^ Only 15 tumors were available for evaluating the percentage of ITV shrinkage before the 5th fraction of SBRT.

## IV. DISCUSSION & CONCLUSIONS

Along with the rapid development of medical linear accelerators, several image methods, including electronic portal image device (EPID), CT on‐rail systems, and CBCT, have been used for determining setup errors.^12^, ^20^, ^21^ Compared with other image methods, CBCT scans can provide volumetric information of lung tumor directly and do not need insertion of metal fiducial markers, which is invasive to the human body.^(22)^ Daily CBCT‐guided radiotherapy allows for assessment of change in volume or position of lung tumors, which further facilitates the formulation of adaptive treatment planning. According to the ICRU 62 Report, PTV is made up of internal organ motion and setup error.^(23)^ Setup error can be different based on the data obtained from each institution. However, organ motion is not completely consistent throughout treatment and tumor volume changes in response to irradiation.^(13)^ Due to the single high dose of SBRT, accurate localization of target volume appears to be especially and extremely crucial. Previous studies shed light on interfractional variation of lung tumor centroid position and volume, but methods employed in treatment simulation procedure and setup procedure were not completely uniform. In addition, the radiotherapy regimens of SBRT in studies were not exactly the same. Results obtained from these studies had slightly heterogeneous distributions. It is exactly based on this consideration that we performed this study. According to the results of our study, a relative high variation of interfractional centroid position was observed in the AP direction during SBRT treatment. The ITV‐PTV margins for anterior‐posterior, superior‐inferior, and left‐right directions in our study theoretically should be 7.9 mm, 6.2 mm, and 5.0 mm, which indicates that an isotropic margin of 5.0 mm might not be sufficient. A phantom study^(18)^ has been performed on the effect of irregular breathing patterns on the ITVs and superior‐inferior lengths delineated on CBCT and 4D CT MIP. According to their results, ITVs delineated on 4D CT MIP and CBCT were reduced by up to 20% and 30% and shortened by up to 7 and 11 mm when irregular motion was introduced. It is indicated that volume was underrepresented at the extremes of motion. Larger treatment margins should be added to the 4D CT MIP ITV for lesions moving greater than 2 cm. In addition, different kinds of breathing patterns may result in different ITVs for the same lesion and sinusoidal pattern represented the ideal clinical scenario. However, our study focused on interfractional variation of the centroid position and volume of ITV during lung SBRT based on bony structure in clinical setting other than phantom study. Not only interfractional variation of SI direction was evaluated, but also AP direction and LR directions were included. In a recent study performed by Mampuya et al.,^(8)^ interfractional variation in lung tumor position with or without abdominal compression during SBRT using CBCT were evaluated. 4D CT was performed under free breathing for all patients, and ITVs were delineated on the MIP using the lung window setting. Treatment planning was made both from 3D CT and 4D CT and lung tumor position was verified using CBCT images. Of all 30 patients, abdominal compression was not used in 14 patients. For this group of patients, the mean±SD of interfractional variation in AP, SI, and LR directions were 0.64±2.69 mm, −0.60±2.10 mm, and 0.17±1.14 mm, respectively. Similar to our result, a slightly higher interfractional variation was observed in the AP direction. In another study performed by Ikushima H et al.,^(21)^ a total of 112 patients with 117 tumors were available for evaluating daily GTV deviations relative to bony references. GTV was delineated on the CT scan obtained through CT on‐rails system before each treatment. Both the free‐breathing helical CT scan and the 4D CT scan were used for treatment planning. All tumors received 40‐50 Gy of SBRT in 4 to 5 fractions using CT on‐rails system. The mean (± SD) daily GTV deviation in all cases was 0.1±4.2 mm (range, −24.1 to 30 mm) in the SI direction, 0.1±3.8 mm (range, −14.1 to 13.6 mm) in the AP direction, and 0.2±2.5 mm (range, −11.7 to 12.7 mm) in the LR direction. No statistically significant trend of GTV position in 3 to 5 consecutively treated fractions was observed. In this study, CT on‐rails system was used for verification. 3D CT scan captures the temporary images of lung tumor which contains only a small part of motion information. This could definitely result in inevitable error when evaluating the GTV deviation. Both 4D CT and CBCT have the advantage of obtaining CT images of lung tumor during several respiratory cycles. However, breathing pattern of patients might change during the treatment course, which not only has impact on the volume of ITVs, but also on the centroid position of ITVs. Results of our study revealed that interfractional variation of ITV centroid position in AP direction was even slightly larger than SI direction. When only bony‐structure‐based setup is performed during SBRT treatment, an isotropic margin of 5 mm around ITV might be not adequate for full coverage of interfractional variation of ITV centroid position.

Additionally, ITV change during radiotherapy treatment may also necessitate the redesigning of radiation planning during treatment. For conventional fractionated radiotherapy for lung tumors, it is routine to shrink the radiation field when irradiated areas including primary lung tumor and the mediastinum receive a radiation dose of 50 Gy.^(24)^ Excessive radiation dose to normal lung tissue will increase the odds of radiation pneumonitis.^(25)^ However, the treatment course of hypofractionated SBRT is usually short and several studies demonstrate no significant volume reduction during SBRT treatment, no matter which kind of image method was used for verification.^26^, ^27^, ^28^ It is reported that significant decrease of tumor volume often occurs after four weeks of SBRT treatment.^(29)^ According to the result repeated in our study, ITV did not decrease significantly from the 1st fraction to the 4th fraction of SBRT, but a time trend was observed when adding the 5th fraction. Out of 37 tumors, only 16 tumors received the 5th fraction of SBRT, which might induce bias when using generalized estimated equation to evaluate the time trend of ITV. Interestingly, ITVs increased slightly before the 2nd and 3rd fraction of SBRT treatment, which might be that the reduction of tumor volume during radiation was offset by tumor radiation‐induced tumor cell swelling or normal tissue inflammation. Thus, more research on the mechanism behind this phenomenon still remains to be conducted. Overall, according to the result of our study, the impact of ITV change during SBRT on the treatment planning is not necessary to be taken into account. However, individualized treatment is highly praised in recent years, so it is of great importance to identify which group of patients would experience great change during SBRT treatment and adaptive radiotherapy should be considered for them. Results in our study showed that only diameter (p=0.000) was found to be significantly associated with the ITV change and data in Tables 2 and 3 showed that large tumors (>3 cm) shrank faster than small tumors (≤3 cm) during SBRT treatment. This offers the possibility of implementing adaptive radiotherapy when relatively large lung tumors are considered for SBRT treatment. This phenomenon needs to be further validated by more research and there still are other potential influence factors to be explored.

Traditionally, the margin of PTV should take respiration motion and setup error into consideration for lung tumors.^(22)^ However, respiratory pattern may differ considerably due to the change of patients’ psychological state or to the concomitant disease. Theoretically, patients would adjust themselves to meet the requirements of treatment with elapsed time. But a single planning CT scan cannot predict the change of respiration pattern.^(30)^ Furthermore, concomitant diseases, such as slight pleural effusion, pneumothorax and pulmonary emphysema, could exert an effect on the position of tumor during treatment. Based on the result of our study, no time trend of 3D vector shift and ITV change were observed and an isotropic margin of 5 mm for ITV‐PTV may be insufficient.

Though both 4D CT and 3D CBCT take the lung motion trajectory into account in order to define ITV, subjective bias in delineating ITV is unavoidable for the limited spatial resolution of 3D CBCT images. Additionally, even translational and rotational movement were made to ensure the optimal matching when registering the 3D CBCT image with AIP image of planning CT, slight bias induced by registration procedure was inevitable, which might influence the ITV centroid position.

Results in our study revealed that internal target volume did not show significant regression during SBRT treatment, but interfractional variation in the ITV centroid position was observed, especially in anterior‐posterior direction. An isotropic margin of 7 mm around ITV might be necessary for adequate coverage of interfractional variation of ITV centroid position using bone structure as reference point. Daily CBCT guided radiotherapy is obligatory for SBRT treatment, especially for those patients whose respiratory pattern is irregular. When necessary, adaptive radiotherapy should be considered for SBRT treatment.

## ACKNOWLEDGMENTS

This research was funded by National Natural Science Foundation of China 81372436; International cooperation project of Henan province 124300510016; the ministry of health and Henan province health department 201201009; scientific and technological project of Henan Scientific and Technological Commission 112102310006; Zhengzhou city science and technology innovation team 121PCXTD524.

## COPYRIGHT

This work is licensed under a Creative Commons Attribution 4.0 International License.
